# Nonthermal Pasteurization of Fermented Green Table Olives by means of High Hydrostatic Pressure Processing

**DOI:** 10.1155/2014/515623

**Published:** 2014-08-18

**Authors:** Anthoula A. Argyri, Efstathios Z. Panagou, George-John E. Nychas, Chrysoula C. Tassou

**Affiliations:** ^1^Hellenic Agricultural Organization-DEMETER, Institute of Technology of Agricultural Products, Sof. Venizelou 1, Lycovrissi, 14123 Athens, Greece; ^2^Department of Food Science and Human Nutrition, Laboratory of Microbiology and Biotechnology of Foods, Agricultural University of Athens, Iera Odos 75, 11855 Athens, Greece

## Abstract

Green fermented olives cv. Halkidiki were subjected to different treatments of high hydrostatic pressure (HHP) processing (400, 450, and 500 MPa for 15 or 30 min). Total viable counts, lactic acid bacteria and yeasts/moulds, and the physicochemical characteristics of the product (pH, colour, and firmness) were monitored right after the treatment and after 7 days of storage at 20^°^C to allow for recovery of injured cells. The treatments at 400 MPa for 15 and 30 min, 450 MPa for 15 and 30 min, and 500 MPa for 15 min were found insufficient as a recovery of the microbiota was observed. The treatment at 500 MPa for 30 min was effective in reducing the olive microbiota below the detection limit of the enumeration method after the treatment and after 1 week of storage and was chosen as being more appropriate for storing olives for an extended time period (5 months). After 5 months of storage at 20^°^C, no microbiota was detected in treated samples, while significant changes for both HHP treated and untreated olives were observed for colour parameters only (minor degradation). In conclusion, HHP treatment may introduce a reliable nonthermal pasteurization method to extend the microbiological shelf-life of fermented table olives.

## 1. Introduction

High hydrostatic pressure (HHP) processing has a great potential in producing high quality foods that are microbiologically safer and with an extended shelf-life [1]. Recently, different HHP treatments have been applied in the food industry on various food products such as meat, fisheries, fruits, and vegetables [2–5]. Due to technological improvement in HPP equipment, industrial application is widespread for a range of pressures between 100 and 800 MPa depending on the desired objective [2]. High pressure is transmitted immediately and uniformly throughout the pressure vessel (Pascal principle) and the process is adiabatic; therefore the food is prevented from being deformed or heated which would modify its quality properties [2, 6, 7].

Table olive processing relies on the microbiota naturally present on fruit surface, processing water and equipment, and in fermentation vessels [8]. Most fermented olives are distributed throughout the market “in bulk” (available in open containers), stored at ambient temperature, and thus exposed to high risk of contamination from the environment [8]. The final product may also be marketed to local markets or exported abroad in glass and plastic containers, as well as in tins, or in other packaging materials such as polyethylene or multilaminated pouches, filled with brine or gases (modified atmospheres) [9, 10]. The latter packages are more convenient to be distributed through the market and at the same time provide added value to the product [10]. However, irrespective of the packaging material, industry usually applies a subsequent thermal pasteurization step to stabilize microbiologically the product [9]. Thermal processing of olives is often associated with quality deterioration, especially if the process is not optimized, resulting in softening of olive tissue, loss of green colour, and development of cooking taste that affects the sensory attributes of the final product [11, 12].

Very limited information is available in the literature about the use of HHP on table olives as an alternative to conventional pasteurization treatment. Pradas et al. [13] studied the effect of HHP on Cornezuelo dressed olives, measuring several physicochemical parameters of HHP treated or untreated olives during storage. The authors reported that no hazardous microorganisms could be enumerated on olives with the exception of yeasts and moulds that were found to be less than 10^6^ CFU/g which is in agreement with the IOC trade standard for table olives [14]. On the other hand, a recent study of Abriouel et al. [11] presented the effect of different levels of HHP and antimicrobials on total viable counts (TVC) and yeasts of Manzanilla Alorena cracked table olives. It was reported that a pressure of 300 MPa for 5 min was effective in reducing yeast population below the detection limit of the enumeration method, but even a pressure of 700 MPa for 5 min was not efficient to suppress the growth of TVC.

According to the above, there is a knowledge gap about the possible HHP treatments that are effective in reducing the population of olive microbiota below the detection limit of the enumeration method, thus guaranteeing an extended shelf life of the product. In this context, the aim of this study was to evaluate the effect of different HHP treatments (combinations of different levels of HHP and processing times) on the indigenous microbiota and the physicochemical parameters of fermented green table olives cv. Halkidiki in an attempt to investigate the efficacy of HHP as an alternative treatment to thermal pasteurization.

## 2. Materials and Methods

### 2.1. Olive Packaging

Green olives cv. Halkidiki from three different batches were obtained from Konstantopoulos S.A. table olive processing industry located in Northern Greece. After the end of the fermentation process, olives were withdrawn from the fermentation vessels and selected by hand to remove defective drupes. Samples (100 g) of fermented green olives were placed in polyethylene pouches, covered with freshly prepared 6% brine (w/v, NaCl) containing 0.2% citric and 0.15% ascorbic acid, and heat sealed. The packages were finally subjected to different HHP treatments as explained below.

### 2.2. Experimental Design

#### 2.2.1. Effect of Different High Pressure Treatments

To investigate the effectiveness of different HHP treatments, inactivation tests were conducted in triplicate at pressures of 400, 450, and 500 MPa for 15 and 30 minutes, respectively. The pressurized packages (HHP samples) were stored at 20°C for 7 days in high precision (0.5°C) incubation chambers (VELP Scientifica, Italy), to allow for recovery of injured cells on the olive fruits and cover brine and thus select the optimum HHP treatment for further storage experiments. Packages without any treatment served as control samples and followed storage at the same conditions as the HHP samples. Microbiological and physicochemical analysis was conducted for both HHP and control samples, at day 0 (right after the HHP treatment) and after 7 days of storage.

#### 2.2.2. Selection of the Most Appropriate HHP Treatment and Storage for 5 Months

Further on, based on the selection of the most effective combination of HHP level and pressurization time, additional packages of green olives were prepared as described above, subjected to HHP processing, and stored for a period of 5 months at 20°C to mimic storage conditions in retail outlets (supermarkets, hypermarkets). Duplicate packages of three different batches of olives were randomly removed and analyzed at preselected time intervals of 0, 7, and 15 days and 1, 2, 3, 4, and 5 months.

### 2.3. High Hydrostatic Pressure (HHP) Treatment

HHP inactivation experiments were conducted in triplicate at pressures of 400, 450, and 500 MPa for 15 and 30 minutes, respectively. Pressurization was carried out at room temperature (18–20°C). The high pressure unit (Food Pressure Unit FPU 1.01, Resato International BV, Roden, Holland) comprised a pressure intensifier and a multivessel system consisting of a central vessel of 250 mL capacity, with a maximum operating pressure and temperature of 1000 MPa and 90°C. The pressure transmitting fluid was polyglycol ISO viscosity class VG 15 (Resato International BV, Roden, Holland). The desired value of pressure was set and, after pressure buildup (20 MPa/s), the pressure vessels were isolated. The pressure of the vessel was released after a preset time interval by opening the corresponding pressure valve. Pressure and temperature were constantly monitored and recorded (in 1 s intervals) during the process [15, 16]. The come-up rate was approximately 100 MPa per 7 sec and the pressure release time was 3 sec. Pressurization time reported in this work does not include the pressure come-up and release times. Further details of the high pressure system and operating conditions can be found elsewhere [17, 18].

### 2.4. Microbiological Analyses

Immediately after the HHP treatment, the enumeration of microorganisms was performed on both olive and brine samples. Specifically, brine samples (1 mL) were aseptically transferred to 9 mL sterile 1/4 Ringer's solution (BR0052G, Oxoid). In the case of olive samples, 10 g of olive flesh was aseptically added to 90 mL sterile 1/4 Ringer's solution and homogenized in a stomacher (Stomacher 400 Circulator, Seward) for 60 s at room temperature. The resulting suspensions were serially diluted in the same diluent and 1 or 0.1 mL samples of the appropriate dilutions were poured or spread on nonselective and selective agar plates. To reduce the detection limit of the enumeration method (for spread plating) to 1 log CFU/g for olive samples and 0 log CFU/mL for brine samples, 1 mL from olive homogenate or 1 mL of brine, respectively, was spread equally on 3 agar plates of each substrate. The selected agar media were the following: Plate Count Agar (CM0325, Oxoid) for total viable counts, incubated at 30°C for 48–72 h; de Man-Rogosa-Sharp (MRS) medium (CM 0361, Oxoid) for LAB, adjusted to pH 5.7 and supplemented with 0.05% (w/v) cycloheximide (Sigma), overlaid with the same medium, and incubated at 30°C for 48–72 h; Rose Bengal Chloramphenicol Agar Base (LAB036 supplemented with selective supplement X009, LAB M) for yeasts/moulds incubated at 25°C for 48–72 h; Violet Red Bile Glucose Agar (CM 0485, Oxoid) for Enterobacteriaceae overlaid with the same medium and incubated at 37°C for 24 h; Pseudomonas agar base** (**CM559 supplemented with selective supplement CFC SR0103, OXOID), for* Pseudomonas* spp. incubated at 25°C for 48 hours. In all growth media incubation time was extended by 1-2 days to allow recovery of lethally injured or stressed cells.

### 2.5. pH Measurement

The pH value of olives and brine was measured with a digital pH meter (HI 2211 pH-ORP Meter, HANNA Instruments, USA). The pH of brine was recorded by immersing the electrode directly in the brine of the package, whereas the pH of olive fruits was measured in the olive homogenate (stomacher homogenate) after the end of the microbiological analysis.

### 2.6. Colour Measurement

The olive colour was assessed by taking at least 10 random measurements from the surface of different olives using a Minolta Chroma Meter fitted with a CR-300 measuring head (Minolta, Osaka, Japan). The CIE (Commission Internationale de l' Eclairage) L*, a*, b* colorimetry system was used for colour determination. L* indicates lightness, and its values range from 0 (an ideal black object) to 100 (an ideal white object). Positive a* values indicate red direction, negative a* value is the green direction, positive b* values are the yellow direction, and negative b* values are the blue direction. The instrument was calibrated with a standard white tile (L* = 96.10, a* = +0.98, and b* = +7.27). At each sampling time, 10 olives from each sample (package) of each different treatment were analyzed in duplicate (2 measurements at random locations on each olive). Thus for each time point and treatment a total of 120 measurements were recorded (3 batches × 2 samples × 20 measurements). Chroma (C*) and hue angle (h*) values were also calculated based on the following equations:

(1) $$\begin{array}{c} C^{*}=\sqrt{a^{*2}+b^{*2}},\\ h^{*}=\tan^{-1}\left(\frac{b^{*}}{a^{*}}\right). \end{array}$$

### 2.7. Firmness Measurement

Firmness of olives was determined using a TA.HD* plus* Texture Analyser equipped with a needle probe and a 50 Kg load cell (Stable Microsystems, Surrey, UK). The speed setting was 30 mm/min, whereas the penetration force was measured in N. At each sampling time, 10 olives from each sample (package) of each different treatment were analyzed. Thus for each time point and each treatment a total of 60 measurements were recorded (3 batches × 2 samples × 10 measurements).

### 2.8. Data Analysis

Each experiment was repeated three times (three different batches of olives) with duplicate samples (packages) opened at each time point. Counts of the different microbial groups were transformed to log CFU/g or log CFU/mL values before computing means and standard deviations. The effects of different treatments on the physicochemical parameters of HHP treated or untreated olives were analyzed using the *t*-test of Excel. Initially, an *F*-test was performed on the dataset to determine if the variances of the tested populations were equal or unequal and in continuance a *t*-test was performed assuming equal or unequal variances, respectively, at 95% confidence interval (*P* < 0.05).

## 3. Results and Discussion

### 3.1. Effect of Different High Pressure Treatments

#### 3.1.1. Effect on the Microbial Community

**Figure 1:**
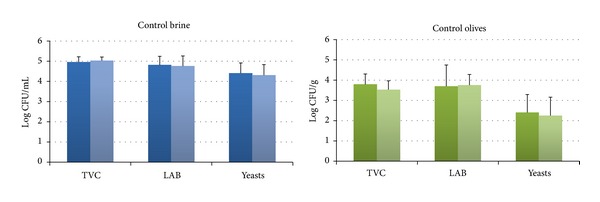
Changes in the population of the indigenous microbiota in brines at day 0 (dark blue) and day 7 (light blue) and in olives at day 0 (dark green) and day 7 (light green) without any HHP treatment. The detection limit of the enumeration method was 0 log CFU/mL for brines and 1 log CFU/g for olives. Data are mean values ± standard deviation of duplicate pouches analyzed from three different batches of olives.

Results showed that the indigenous microbiota of olives prior to treatment comprised LAB followed by yeasts (Figure 1). The initial mean population of LAB was 4.81 ± 0.43 log CFU/mL and 3.70 ± 1.05 log CFU/g in the brines and olives, respectively, whereas the corresponding population of yeasts was 4.04 ± 1.31 log CFU/mL in the brines and 2.40 ± 0.89 log CFU/g in olives (Figure 1). These results are within the expected range of LAB and yeasts reported previously for fermented black or green olives with or without covering brines [12, 19–21]. The microbial population showed no changes during storage at 20°C for 7 days for the control olive samples (Figure 1).* Pseudomonas* spp. and enterobacteria were not detected at any stage of the above storage period for both HHP treated and control samples.

**Figure 2:**
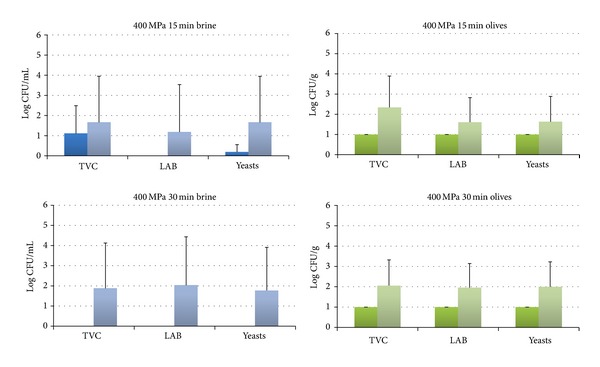
Changes in the population of the indigenous microbiota in brines at day 0 (dark blue) and day 7 (light blue) and in olives at day 0 (dark green) and day 7 (light green) treated at 400 MPa for 15 or 30 min. The detection limit of the enumeration method was 0 log CFU/mL for brines and 1 log CFU/g for olives. Data are mean values ± standard deviation of duplicate pouches analyzed from three different batches of olives.

**Figure 3:**
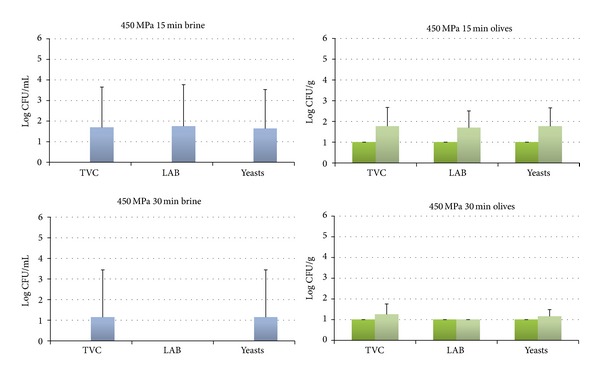
Changes in the population of the indigenous microbiota in brines at day 0 (dark blue) and day 7 (light blue) and in olives at day 0 (dark green) and day 7 (light green) treated at 450 MPa for 15 or 30 min. The detection limit of the enumeration method was 0 log CFU/mL for brines and 1 log CFU/g for olives. Data are mean values ± standard deviation of duplicate pouches analyzed from three different batches of olives.

**Figure 4:**
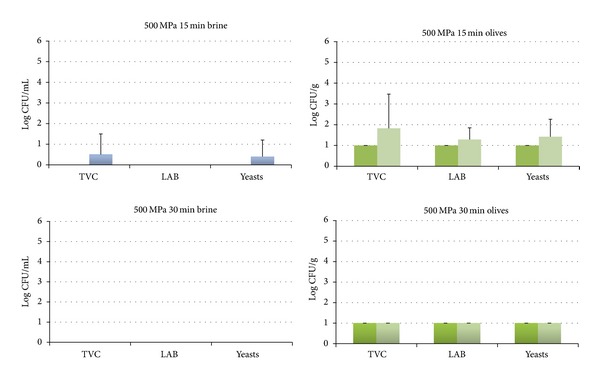
Changes in the population of the indigenous microbiota in brines at day 0 (dark blue) and day 7 (light blue) and in olives at day 0 (dark green) and day 7 (light green) treated at 500 MPa for 15 or 30 min. The detection limit of the enumeration method was 0 log CFU/mL for brines and 1 log CFU/g for olives. Data are mean values ± standard deviation of duplicate pouches analyzed from three different batches of olives.

The HHP treatment resulted in the reduction of the microbial populations in both brines and olives below the detection limit of the enumeration method in all cases right after the treatment (Figures 2–4), with the exception of 400 MPa for 15 min where a recovery of yeasts was observed in the brines (Figure 2). The subsequent storage of the HHP treated samples for 7 days at 20°C resulted in the recovery of LAB and yeasts in all studied treatments except from 500 MPa for 30 min where no growth of LAB and yeasts was observed (Figures 2–4). More specifically, LAB were recovered in both brines and olives in treatments of 400 MPa for 15 and 30 min (Figure 2) and 450 MPa for 15 min (Figure 3) as well as in olives at 450 MPa for 15 min (Figure 3). Yeasts were found to be more resistant than LAB and were recovered in all cases except 500 MPa for 30 min (Figures 2–4). These findings are in contrast with a previous study of Abriouel et al. [11] who reported no viable yeast counts in Manzanilla Aloreña cracked olives at pressures of 300 MPa or higher for 5 min. On the other hand, the same authors have shown that even a pressure of 700 MPa for 5 min was not capable of reducing the bacteria below the detection limit that were found to be more HHP resistant than yeasts. Sánchez et al. [22] reported that a pressure of 450 MPa for 10 min was not sufficient to reduce bacteria and yeasts/moulds in olive paste below the detection limit, whereas a treatment of 600 MPa for 5 or 10 min was effective against yeasts/moulds but not bacteria.

Thus, according to the findings of this work, the treatment at 500 MPa for 30 min was chosen as the most suitable condition of pressure/time to study the effect of the specific HHP treatment on the storage of green table olives.

#### 3.1.2. Effect on the Physicochemical Parameters

The initial pH values in olive samples prior to pressurization were found to be 3.97 ± 0.07 and 4.12 ± 0.08 in brine and olives, respectively, and did not change significantly after 1 week. Similar values were observed for the HHP treated samples (Table 1).

The HHP processing was found to reduce significantly the L* value of the samples right after treatment at 500 MPa for 30 min in comparison with the control samples, indicating slightly darker olive products due to HHP processing. Moreover, the L* values decreased and a* values increased after 1-week storage for HHP samples in all treatments except from the case of 400 MPa for 15 min (Table 1). Increasing a* values during 7-day storage indicate an increase in the red component of the colour of olives. The HHP effect on the b* values was a reduction (*P* < 0.05) in samples treated for 30 min irrespective of the pressure applied. However, no further reduction was observed after 1 week of storage. On the contrary, all the samples treated for 15 min at all pressure levels did not show reduced b* values right after the treatment, but a slight reduction was observed after 7 days indicating a minor loss in the yellow tonalities (Table 1).

Finally, no significant effect (*P* < 0.05) on the firmness of the HHP samples was observed neither after the treatment, nor after 7 days of storage.

### 3.2. Effect of 500 MPa for 30 min on the Storage of Olives

#### 3.2.1. Effect on the Microbial Evolution

HHP treatment at 500 MPa for 30 min resulted in the reduction of all the indigenous microbiota below the detection limit of the enumeration method. Moreover, no growth was observed during storage for 5 months at 20°C. Regarding the control (unpressurized) samples, only minor changes in the population of LAB and yeasts were observed after 5 months of storage (data not shown). The initial mean population of LAB was 4.98 ± 0.28 log CFU/mL and 4.53 ± 0.34 log CFU/g in the brines and olives, respectively, whereas the corresponding population of yeasts was 4.06 ± 0.77 log CFU/mL in the brines and 2.68 ± 0.73 log CFU/g in olives. The final population of LAB was 5.10 ± 0.11 log CFU/mL and 4.48 ± 0.42 log CFU/g in brines and olives, respectively, whereas the corresponding population of yeasts was 4.01 ± 0.65 log CFU/mL in brines and 3.22 ± 0.24 log CFU/g in olives. No* Pseudomonas* spp. or enterobacteria were detected at any stage of the storage period in control or HHP treated samples.

#### 3.2.2. Effect on the Physicochemical Parameters

Statistically significant changes during storage of both HHP treated and control samples were observed only for colour parameters. The remainder of the studied parameters (firmness and pH) did not show any significant changes (Table 2). These results are in contrast with a previous study of Pradas et al. [13] where softening of olives and decrease in pH were reported during storage for both HHP treated and untreated Cornezuelo dressed olives. This could be attributed to the fact that these olives were not previously fermented when subjected to HHP treatment but they were packed in brine and dressed with sodium chloride, vinegar, and various herbs (thyme, garlic, and fennel).

Colour has a key contribution in the marketability of green table olives as a vivid green colour is an essential characteristic of the product, especially in Spanish-style processing [23]. The lightness (L*) was fairly high at the beginning of storage and showed a slight decrease at all cases. The a* values were initially negative indicating green tonalities. During storage however, a gradual decrease was observed in both control and HHP samples indicating a slight decrease in the green olive colour. Finally, the values of b* were also positive (yellow) at day 0 and showed a decrease, indicating a decrease in olive yellowness (Table 2).

**Figure 5:**
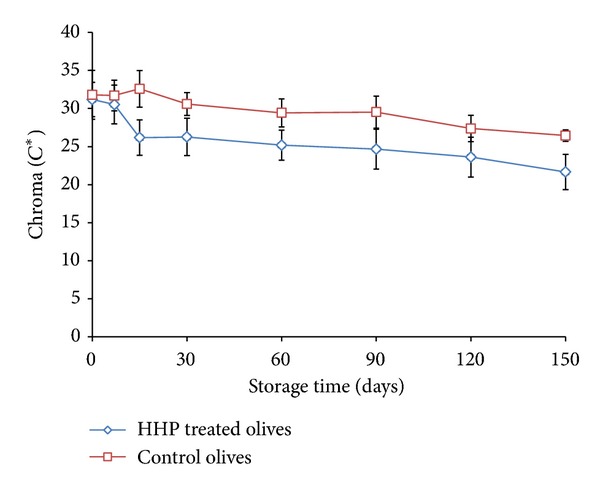
Changes in chroma (C*) values of HHP treated and control olives during storage for 5 months at 20°C. Data are mean values ± standard deviation of duplicate pouches analyzed from three different batches of olives.

Regarding the control samples, the L* and b* values were found to decrease during storage, with statistically significant reduction being observed after the 1st month. The a* values were found to increase during storage, with statistically significant reduction being observed after the 2nd month of storage (Table 2). Concerning the HHP treated samples, the changes for all colour parameters were observed earlier in comparison with the control (i.e., at the 15th day of storage) and were more intense (Table 2). Moreover, at the last month of storage of HHP treated samples, the a* value was found to be positive indicating a light browning of olives. A gradual decrease in chroma (C*) values was observed in both HHP treated and control olive samples throughout storage (Figure 5) that was higher in pressure treated olives (*ca.* 10 units) compared to control samples (*ca.* 5 units), indicating slightly higher colour intensity in untreated samples. Concerning hue angle values (h*), a slight increase of 4-5° towards yellow colour (hue angle 90°) was recorded with no significant difference between the applied treatments (data not shown). Similar results have been reported by Pradas et al. [13] for a moderate degradation of the colour in both HHP treated and untreated samples, with no further details given about the differences between the treatments.

A possible solution for further improving the colour in HHP treated olives is the addition of ascorbic acid, which was shown to enhance colour of Manzanilla Aloreña cracked table olives [24]. In the latter study, the highest concentration of ascorbic acid that was also shown to have the best effect on colour was 15 g/L. Since in this study the initial concentration of ascorbic acid added in the brines was 1.5 g/L, a future increase in the concentration of the acid could improve the colour maintenance of olives during storage.

## 4. Conclusions

In conclusion, the HHP treatment was applied to extend the microbiological shelf-life and the quality of green table olives. The treatment at 500 MPa for 30 min can significantly extend the shelf-life of these products, since it was found efficient at reducing the indigenous microbiota below the detection limit. Additionally, no microbial growth was observed after storage of the HHP treated olives at 20°C for 5 months. Thus, HHP processing may introduce a reliable nonthermal method to extend the shelf-life of fermented green table olives. However, additional research is needed in order to establish HHP processing as a useful tool for the table olive industry.

## Figures and Tables

**Table 1:** Evolution of the physicochemical parameters of olives pressurized or not (control) at 400, 450, or 500 MPa for 15 or 30 min and stored at 20°C for 7 days.

Pressure (MPa)	Processing time (min)	Colour					
		L* value		a* value		b* value	
		0 days	7 days	0 days	7 days	0 days	7 days
Control (unpressurized)	—	53.95 ± 2.44^a,x^	54.71 ± 1.64^a,x^	−4.17 ± 0.70^a,x^	−4.43 ± 1.01^a,x^	31.53 ± 2.67^a,x^	32.54 ± 2.29^a,x^
400	15	53.84 ± 2.50^a,x^	53.02 ± 1.81^b,x^	−4.66 ± 0.66^a,x^	−4.17 ± 0.74^a,b,x^	31.50 ± 2.86^a,x^	29.89 ± 2.02^b,y^
	30	53.97 ± 1.45^a,x^	51.87 ± 1.70^b,c,y^	−4.58 ± 0.66^a,x^	−3.86 ± 0.76^b,c,y^	30.55 ± 2.22^b,x^	29.25 ± 1.93^b,x^
450	15	54.28 ± 3.09^a,x^	51.95 ± 1.89^b,c,y^	−3.96 ± 0.71^a,x^	−3.28 ± 0.87^c,y^	31.59 ± 3.76^a,x^	29.44 ± 1.83^b,y^
	30	53.85 ± 3.46^a,x^	52.75 ± 1.97^b,c,x,y^	−3.90 ± 0.68^a,x^	−3.23 ± 0.79^c,y^	30.89 ± 3.25^b,x^	29.66 ± 1.75^b,y^
500	15	53.12 ± 2.58^a,x^	51.91 ± 2.04^b,c,y^	−4.34 ± 0.65^a,x^	−3.39 ± 0.61^c,y^	31.23 ± 2.36^a,x^	30.08 ± 1.72^b,y^
	30	52.01 ± 3.07^b,x^	50.71 ± 2.77^b,c,y^	−4.22 ± 1.26^a,x^	−3.13 ± 0.44^c,y^	30.79 ± 2.17^b,x^	30.22 ± 1.30^b,x^

		Firmness (Nt)		pH			
				Brine		Olives	
		0 days	7 days	0 days	7 days	0 days	7 days

Control (unpressurized)	—	4.24 ± 0.94^a,x^	4.42 ± 1.08^a,x^	3.97 ± 0.07^a,x^	3.99 ± 0.08^a,x^	4.12 ± 0.08^a,x^	4.10 ± 0.08^a,x^
400	15	4.23 ± 0.91^a,x^	4.15 ± 1.48^a,x^	3.99 ± 0.04^a,x^	4.02 ± 0.06^a,x^	4.14 ± 0.06^a,x^	4.11 ± 0.04^a,x^
	30	4.89 ± 1.03^a,x^	4.14 ± 1.42^a,x^	3.88 ± 0.16^a,x^	4.09 ± 0.06^a,x^	4.18 ± 0.01^a,x^	4.16 ± 0.01^a,x^
450	15	4.50 ± 0.85^a,x^	4.61 ± 1.08^a,x^	3.90 ± 0.01^a,x^	3.87 ± 0.04^a,x^	4.01 ± 0.15^a,x^	4.04 ± 0.03^a,x^
	30	4.92 ± 0.91^a,x^	4.94 ± 0.97^a,x^	3.93 ± 0.01^a,x^	3.90 ± 0.01^a,x^	4.11 ± 0.09^a,x^	4.04 ± 0.00^a,x^
500	15	4.25 ± 1.05^a,x^	4.07 ± 1.12^a,x^	4.04 ± 0.09^a,x^	4.04 ± 0.07^a,x^	4.12 ± 0.06^a,x^	4.10 ± 0.09^a,x^
	30	4.75 ± 1.29^a,x^	4.77 ± 1.17^a,x^	4.07 ± 0.04^a,x^	4.02 ± 0.02^a,x^	4.15 ± 0.02^a,x^	4.12 ± 0.04^a,x^

^a,b,c^Different letters within the same column indicate significant differences between different treatments at the specific storage time (*P* < 0.05).

^x,y^Different letters within the same row indicate significant differences between 0th and 7th day at the same treatment (*P* < 0.05).

**Table 2:** Evolution of the physicochemical parameters of olives pressurized or not (control) at 500 MPa for 30 min and stored at 20°C for 5 months.

	0 days	7 days	15 days	1 month	2 months	3 months	4 months	5 months
500 MPa								
*L*	52.05 ± 1.31^a*^	51.89 ± 2.69^a^	48.17 ± 1.57^b^	48.32 ± 2.11^b^	47.27 ± 2.04^c^	47.18 ± 2.16^c^	45.98 ± 1.17^d^	44.92 ± 1.49^e^
*a*	−3.85 ± 0.62^a^	− 3.82 ± 0.79^a^	− 1.75 ± 0.94^b^	− 2.07 ± 0.82^b^	− 1.77 ± 0.60^b,c^	− 1.50 ± 0.77^c^	− 0.61 ± 0.76^d^	0.67 ± 0.52^d^
*b*	30.95 ± 2.17^a^	30.29 ± 2.43^a^	26.12 ± 2.13^b^	26.18 ± 2.32^b^	25.12 ± 1.88^b,c^	24.62 ± 2.50^c,d^	23.61 ± 2.51^d^	21.65 ± 2.25^e^
Firmness	4.27 ± 1.10^a^	3.88 ± 1.23^a^	3.45 ± 0.96^a^	3.93 ± 1.37^a^	3.65 ± 1.06^a^	3.66 ± 1.05^a^	3.72 ± 1.22^a^	3.77 ± 0.94^a^
pH brine	3.94 ± 0.17^a^	3.94 ± 0.07^a^	4.03 ± 0.16^a^	4.14 ± 0.01^a^	4.07 ± 0.04^a^	4.10 ± 0.03^a^	3.86 ± 0.02^a^	3.86 ± 0.01^a^
pH olives	4.18 ± 0.11^a^	4.11 ± 0.08^a^	4.17 ± 0.04^a^	4.18 ± 0.01^a^	4.17 ± 0.01^a^	4.18 ± 0.06^a^	4.13 ± 0.06^a^	4.19 ± 0.02^a^
Control								
*L*	52.94 ± 2.24^x^	52.69 ± 1.68^x^	53.14 ± 2.52^x^	51.94 ± 2.24^y^	51.01 ± 2.65^y^	48.47 ± 2.17^z^	47.63 ± 1.89^z^	47.79 ± 1.34^z^
*a*	−3.78 ± 0.97^x^	− 3.72 ± 0.72^x^	−3.78 ± 1.23^x^	− 3.77 ± 0.59^x,y^	−2.84 ± 1.29^y^	−2.27 ± 1.05^y^	−2.26 ± 0.53^y^	−1.22 ± 0.21^z^
*b*	31.57 ± 3.06^x^	31.49 ± 1.89^x^	32.37 ± 2.05^x^	30.36 ± 1.39^y^	29.29 ± 1.33^y^	29.44 ± 1.83^y^	27.28 ± 1.64^z^	26.42 ± 0.72^z^
Firmness	4.24 ± 0.91^x^	4.46 ± 1.07^x^	4.34 ± 1.09^x^	4.39 ± 1.09^x^	4.42 ± 1.08^x^	4.29 ± 0.93^x^	4.24 ± 0.94^x^	4.20 ± 1.03^x^
pH brine	3.90 ± 0.20^x^	3.99 ± 0.06^x^	3.98 ± 0.11^x^	4.03 ± 0.07^x^	3.89 ± 0.09^x^	4.03 ± 0.01^x^	4.08 ± 0.03^x^	3.93 ± 0.09^x^
pH olives	4.18 ± 0.01^x^	4.10 ± 0.03^x^	4.12 ± 0.08^x^	4.18 ± 0.01^x^	4.07 ± 0.07^x^	4.16 ± 0.02^x^	4.06 ± 0.03^x^	4.13 ± 0.06^x^

∗Different letters within the same row indicate significant differences between each storage time (*P* < 0.05).

## References

[1] Patterson MF (2005). Microbiology of pressure-treated foods. *Journal of Applied Microbiology*.

[2] Rendueles E, Omer MK, Alvseike O, Alonso-Calleja C, Capita R, Prieto M (2011). Microbiological food safety assessment of high hydrostatic pressure processing: a review. *LWT—Food Science and Technology*.

[3] Farr D (1990). High pressure technology in the food industry. *Trends in Food Science and Technology*.

[4] Devlieghere F, Vermeiren L, Debevere J (2004). New preservation technologies: possibilities and limitations. *International Dairy Journal*.

[5] Considine KM, Kelly AL, Fitzgerald GF, Hill C, Sleator RD (2008). High-pressure processing—effects on microbial food safety and food quality. *FEMS Microbiology Letters*.

[6] Smelt JP (1998). Recent advances in the microbiology of high pressure processing. *Trends in Food Science and Technology*.

[7] Wilson DR, Dabrowski L, Stringer S, Moezelaar R, Brocklehurst TF (2008). High pressure in combination with elevated temperature as a method for the sterilisation of food. *Trends in Food Science and Technology*.

[8] Panagou EZ, Nychas GE, Sofos JN (2013). Types of traditional Greek foods and their safety. *Food Control*.

[9] Sánchez AH, Montaño A, Rejano L (1997). Effect of preservation treatment, light, and storage time on quality parameters of Spanish-style green olives. *Journal of Agricultural and Food Chemistry*.

[10] Doulgeraki AI, Hondrodimou O, Iliopoulos V, Panagou EZ (2012). Lactic acid bacteria and yeast heterogeneity during aerobic and modified atmosphere packaging storage of natural black Conservolea olives in polyethylene pouches. *Food Control*.

[11] Abriouel H, Benomar N, Gálvez A, Pérez-Pulido R, Gálvez A (2014). Preservation of Manzanilla Alore±a cracked green table olives by high hydrostatic pressure treatments singly or in combination with natural antimicrobials. *LWT—Food Science and Technology*.

[12] Dimou A, Panagou E, Stoforos NG, Yanniotis S (2013). Analysis of thermal processing of table olives using computational dynamics. *Journal of Food Science*.

[13] Pradas I, del Pino B, Peña F (2012). The use of high hydrostatic pressure (HHP) treatments for table olives preservation. *Innovative Food Science and Emerging Technologies*.

[14] International Olive Council (IOC) (2004). *Trade Standard Applying to Table Olives*.

[15] Katsaros GI, Katapodis P, Taoukis PS (2009). High hydrostatic pressure inactivation kinetics of the plant proteases ficin and papain. *Journal of Food Engineering*.

[16] Katsaros GI, Katapodis P, Taoukis PS (2009). Modeling the effect of temperature and high hydrostatic pressure on the proteolytic activity of kiwi fruit juice. *Journal of Food Engineering*.

[17] Panagou EZ, Tassou CC, Manitsa C, Mallidis CG (2007). Modelling the effect of high pressure on the inactivation kinetics of a pressure-resistant strain of *Pediococcus damnosus* in phosphate buffer and gilt-head seabream (*Sparus aurata*). *Journal of Applied Microbiology*.

[18] Tassou CC, Panagou EZ, Samaras FJ, Galiatsatou P, Mallidis CG (2008). Temperature-assisted high hydrostatic pressure inactivation of *Staphylococcus aureus* in a ham model system: evaluation in selective and nonselective medium. *Journal of Applied Microbiology*.

[19] Panagou EZ (2004). Effect of different packing treatments on the microbiological and physicochemical characteristics of untreated green olives of the Conservolea cultivar. *Journal of the Science of Food and Agriculture*.

[20] Arroyo-López FN, Romero C, Durán Quintana MC, López López A, García García P, Garrido Fernández A (2005). Kinetic study of the physicochemical and microbiological changes in “seasoned” olives during the shelf-life period. *Journal of Agricultural and Food Chemistry*.

[21] Arroyo López FN, Quintana MCD, Fernández AG (2007). Modelling of the growth-no growth interface of Issatchenkia occidentalis, an olive spoiling yeast, as a function of the culture media, NaCl, citric and sorbic acid concentrations: study of its inactivation in the no growth region. *International Journal of Food Microbiology*.

[22] Sánchez J, De Miguel C, Ramírez MR, Delgado J, Franco MN, Martín D (2012). Effect of high hydrostatic pressure versus thermal pasteurization on the microbiological, sensory aspects and oxidative stability of olive pate. *Grasas y Aceites*.

[23] Sánchez-Gómez AH, García-García P, Garrido-Fernández A (2013). Spanish-style green table olive shelf-life. *International Journal of Food Science & Technology*.

[24] Arroyo-López FN, Bautista-Gallego J, Durán-Quintana MC, Garrido-Fernández A (2008). Effects of ascorbic acid, sodium metabisulfite and sodium chloride on freshness retention and microbial growth during the storage of Manzanilla-Aloreña cracked table olives. *LWT—Food Science and Technology*.

